# Clinical and Genetic Findings in a Series of Eight Families with Arthrogryposis

**DOI:** 10.3390/genes13010029

**Published:** 2021-12-23

**Authors:** Marzia Pollazzon, Stefano Giuseppe Caraffi, Silvia Faccioli, Simonetta Rosato, Heidi Fodstad, Belinda Campos-Xavier, Emanuele Soncini, Giuseppina Comitini, Daniele Frattini, Teresa Grimaldi, Maria Marinelli, Davide Martorana, Antonio Percesepe, Silvia Sassi, Carlo Fusco, Giancarlo Gargano, Andrea Superti-Furga, Livia Garavelli

**Affiliations:** 1 Medical Genetics Unit, Azienda USL-IRCCS di Reggio Emilia, 42123 Reggio Emilia, Italy; marzia.pollazzon@ausl.re.it (M.P.); stefanogiuseppe.caraffi@ausl.re.it (S.G.C.); simonetta.rosato@ausl.re.it (S.R.); maria.marinelli@ausl.re.it (M.M.); 2 Rehabilitation Pediatric Unit, Azienda USL-IRCCS of Reggio Emilia, 42123 Reggio Emilia, Italy; silvia.faccioli@ausl.re.it (S.F.); silvia.sassi@ausl.re.it (S.S.); 3 PhD Program in Clinical and Experimental Medicine, Department of Biomedical, Metabolic and Neural Sciences, University of Modena and Reggio Emilia, 41125 Modena, Italy; 4 Division of Genetic Medicine, Lausanne University Hospital (CHUV), University of Lausanne, 1011 Lausanne, Switzerland; Heidi.Fodstad@chuv.ch (H.F.); Belinda.Xavier@chuv.ch (B.C.-X.); asuperti@unil.ch (A.S.-F.); 5 Department of Obstetrics & Gynaecology, Ospedale di Sassuolo, 41049 Sassuolo, Italy; emanuele.soncini@outlook.it; 6 Department of Obstetrics & Gynaecology, Azienda USL-IRCCS di Reggio Emilia, 42123 Reggio Emilia, Italy; giuseppina.comitini@ausl.re.it; 7 Child Neurology and Psychiatry Unit, Azienda USL-IRCCS di Reggio Emilia, 42123 Reggio Emilia, Italy; daniele.frattini@ausl.re.it (D.F.); carlo.fusco@ausl.re.it (C.F.); 8 Department of Cardiology, Azienda USL-IRCCS di Reggio Emilia, 42123 Reggio Emilia, Italy; teresa.grimaldi@ausl.re.it; 9 Medical Genetics, Department of Medicine and Surgery, University of Parma, 43126 Parma, Italy; dmartorana@ao.pr.it (D.M.); antonio.percesepe@unipr.it (A.P.); 10 Neonatal Intensive Care Unit, Azienda USL-IRCCS di Reggio Emilia, 42123 Reggio Emilia, Italy; giancarlo.gargano@ausl.re.it

**Keywords:** arthrogryposis, distal arthrogryposis, multiple pterygium syndrome (MPS), Escobar syndrome, amyoplasia, genetic testing, differential diagnosis, prognosis

## Abstract

The term “arthrogryposis” is used to indicate multiple congenital contractures affecting two or more areas of the body. Arthrogryposis is the consequence of an impairment of embryofetal neuromuscular function and development. The causes of arthrogryposis are multiple, and in newborns, it is difficult to predict the molecular defect as well as the clinical evolution just based on clinical findings. We studied a consecutive series of 13 participants who had amyoplasia, distal arthrogryposis (DA), or syndromic forms of arthrogryposis with normal intellectual development and other motor abilities. The underlying pathogenic variants were identified in 11 out of 13 participants. Correlating the genotype with the clinical features indicated that prenatal findings were specific for DA; this was helpful to identify familial cases, but features were non-specific for the involved gene. Perinatal clinical findings were similar among the participants, except for amyoplasia. Dilatation of the aortic root led to the diagnosis of Loeys–Dietz syndrome (LDS) in one case. The phenotype of DA type 5D (DA5D) and Escobar syndrome became more characteristic at later ages due to more pronounced pterygia. Follow-up indicated that DA type 1 (DA1)/DA type 2B (DA2B) spectrum and LDS had a more favorable course than the other forms. Hand clenching and talipes equinovarus/rocker bottom foot showed an improvement in all participants, and adducted thumb resolved in all forms except in amyoplasia. The combination of clinical evaluation with Next Generation Sequencing (NGS) analysis in the newborn may allow for an early diagnosis and, particularly in the DAs, suggests a favorable prognosis.

## 1. Introduction

Congenital contractures can be divided into two groups: isolated and multiple. Isolated contractures affect only one area of the body, e.g., congenital talipes equinovarus, found in approximately 1 in 500 live births [1,2]. 

Multiple congenital contractures affecting two or more areas of the body are often indicated with the term arthrogryposis. This is not a specific diagnosis, but rather a clinical finding, reported in over 400 syndromes [3,4]. Overall, the prevalence of arthrogryposis is 1 in 3000 live births [5]. The different characteristics of the various disorders, as well as their differences in mode of inheritance, course, treatment guidelines, and prognosis, underline the importance of making a specific diagnosis in each patient [6,7,8,9,10,11]. Achieving a molecular diagnosis has positive psychological repercussions on the patient’s family, as well as economic consequences for the healthcare system as it prevents the execution of inappropriate investigations and therapies. Since arthrogryposis is a congenital pathology, the clinical diagnosis is generally made at birth or in the neonatal period. Some pregnancies may show a reduction in the amount of amniotic fluid, a reduction in fetal movements, and/or contractures on prenatal ultrasound. Decreased movements can perturb molecular mechanisms and signaling pathways involved in the formation of joints during development [12].

In the wide field of differential diagnosis, it is important to define the neurological functionality of the patient. A heterogeneous group of disorders exists in which multiple congenital contractures are present at birth, but other central neurological functions such as intellectual development and motor abilities are normal. These include: amyoplasia, distal arthrogryposis (DA), metabolic disorders, generalized connective tissue disorder, skeletal dysplasia or other syndromic conditions, intrauterine space limitations (twin pregnancy), vascular uterine impairment, maternal pathology, or therapy in pregnancy. Conversely, an altered neurological examination indicates that movements in the uterus have decreased due to an alteration of the central nervous system (CNS), peripheral nervous system (PNS), neuromuscular junction or muscle [13]. We have focused our attention on participants with normal neurological functions.

Amyoplasia is characterized by a significant congenital reduction in muscle development and has peculiar clinical features: shoulder rotation and adduction, elbow extension, flexion and ulnar deviation of the wrists, rigid fingers, and thumbs positioned in the palm of the hand. The hips can be dislocated, the knees are generally extended, and the feet have severe equinovarus contractures. The etiology of the disease is not yet known, but an epigenetic disease or a vascular origin have been hypothesized [14]. Most of the described cases are sporadic, but there are also rare cases of potentially familial amyoplasia [15].

DA represents a group of conditions that mainly involve the distal parts of the limbs. About 50% of cases are associated with variants in genes coding for contractile proteins of the skeletal muscle: *FBN2*, *MYBPC1*, *MYH3*, *MYH8*, *PIEZO2*, *TNNI2*, *TNNT3*, and *TPM2*. Inheritance usually follows an autosomal dominant mode, and clinical expression is highly variable, including incomplete penetrance. At least 19 types of DA have been described [3,4,16,17]. Biallelic variants in the *ECEL1* gene have been found in DA type 5D (DA5D), with a characteristic phenotype: severe camptodactyly of the hands, milder camptodactyly in the feet, limited hip flexion, scoliosis, ocular findings (eyelid ptosis, ophthalmoplegia, astigmatism), and distinctive facial features (microretrognathia, grooved tongue) [18,19]. More recently, a new intronic variant and a novel deletion in the gene were described [20,21].

Syndromic forms are widely heterogeneous, with variable course and prognosis depending on the clinical problems associated with arthrogryposis. A noteworthy subgroup is represented by multiple pterygium syndrome (MPS), characterized by multiple pterygium, scoliosis, and congenital contractures of the limbs. Non-lethal (Escobar syndrome) and lethal forms, with both autosomal dominant and recessive modes of inheritance, have been described. Variants in the *CHRNG* gene are associated with all four forms, while variants in the *CHRND* and *CHRNA1* genes are responsible for lethal forms [22].

Next generation sequencing (NGS) techniques, including gene panels, whole exome sequencing (WES), and whole genome sequencing (WGS), allow the identification of new genes associated with arthrogryposis and often clinical diagnosis is only possible once such investigations have been concluded (reverse genetics) [23,24,25]. Currently, there are 402 genes thought to be associated with this condition [25]. In a recent study, WES analysis was extended to a cohort of 89 families, and the diagnosis was reached in 65% of cases, with 19% having potentially pathogenetic variants in more than one locus. In another study, disease gene identification was achieved through WES in 52.7% of index patients [26]. The hypothesis of an oligogenic inheritance for different forms of arthrogryposis has therefore been proposed [27]. 

In this study, we have re-evaluated the data relating to participants who were offered genetic counseling at our institution, in whom arthrogryposis with otherwise normal neurological development was diagnosed. The contribution of our work is represented by the detailed clinical and instrumental findings with periodic follow-up and extensive photographic documentation provided, paralleled by in-depth genetic investigations. A limitation of our study, in consideration of the small number of cases, concerns the difficulty of carrying out genotype–phenotype correlations, which, however, have been proven complicated even in studies with extended cohorts.

## 2. Materials and Methods

### 2.1. Study Subjects

A group of 13 participants (pts) from 8 unrelated families suffering from arthrogryposis with normal neurological development, all in follow-up at the Medical Genetics Unit of Azienda USL- IRCCS di Reggio Emilia, Italy, were enrolled in this study. Three participants had a Roma ethnic background, one patient had mixed origins (paternal grandparents from the Dominican Republic, maternal grandparents of Italian origins), and the rest had Italian origins. Each patient had previously undergone genetic and dysmorphological evaluations at different ages, cytogenetic analysis, and molecular genetic testing. In the present study, we re-evaluated these pre-existing participants’ data in order to describe our cohort. Signed informed consent for inclusion in this study was provided by all subjects of at least 18 years of age, or by both parents for participants under the age of 18. The study was conducted in accordance with the Declaration of Helsinki, and the protocol was approved by the Ethics Committee of AVEN (Project identification code 722/2020).

### 2.2. Genetic Analysis

Prenatal genetic testing reported in 4 cases had already been carried out at the time of the first clinical encounter. Postnatal cytogenetic analyses were performed at the Laboratory of Genetics of Azienda USL- IRCCS di Reggio Emilia, Italy, in accordance with the approved clinical guidelines at the time of testing. Further molecular testing was performed at the Division of Genetic Medicine of Lausanne University Hospital, Switzerland. In pts 4 and 12, karyotype analysis on peripheral blood was performed. In pt 13, karyotype on blood and fibroblasts, FISH for 7q11.23 region, FISH for subtelomeric regions, FISH for chromosome 8 on fibroblasts, and whole exome sequencing (WES) were performed. In pt 9, molecular analysis of the *FLNB* gene was performed before specific analysis for arthrogryposis. In pt 10, CGH-array (60K, Illumina, San Diego, CA, USA) and molecular analysis of the *DMPK* gene were performed. NGS analysis of a panel of genes responsible for arthrogryposis was performed in seven participants using the TruSight or TruSight One Expanded kit from Illumina with sequencing on the MiSeq or NextSeq system. In pt 11, an NGS panel for genes associated with aortopathies was also performed, due to the suspicion of Loeys-Dietz syndrome (LDS). In pts 9 and 13, WES analysis filtered for specific genes was performed. Variants were classified according to the recommendations of the American College of Medical Genetics (ACMG) [28] (Table S1). Bidirectional Sanger sequencing was employed for confirmation of all disease-causing variants detected by NGS and subsequent co-segregation analysis in affected family members. 

## 3. Results

### 3.1. Participants and Clinical Information

**Figure 1:**
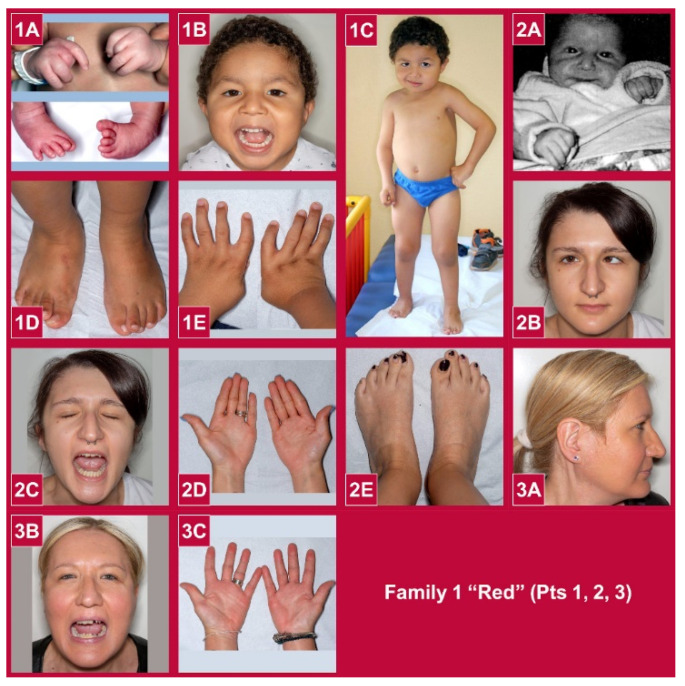

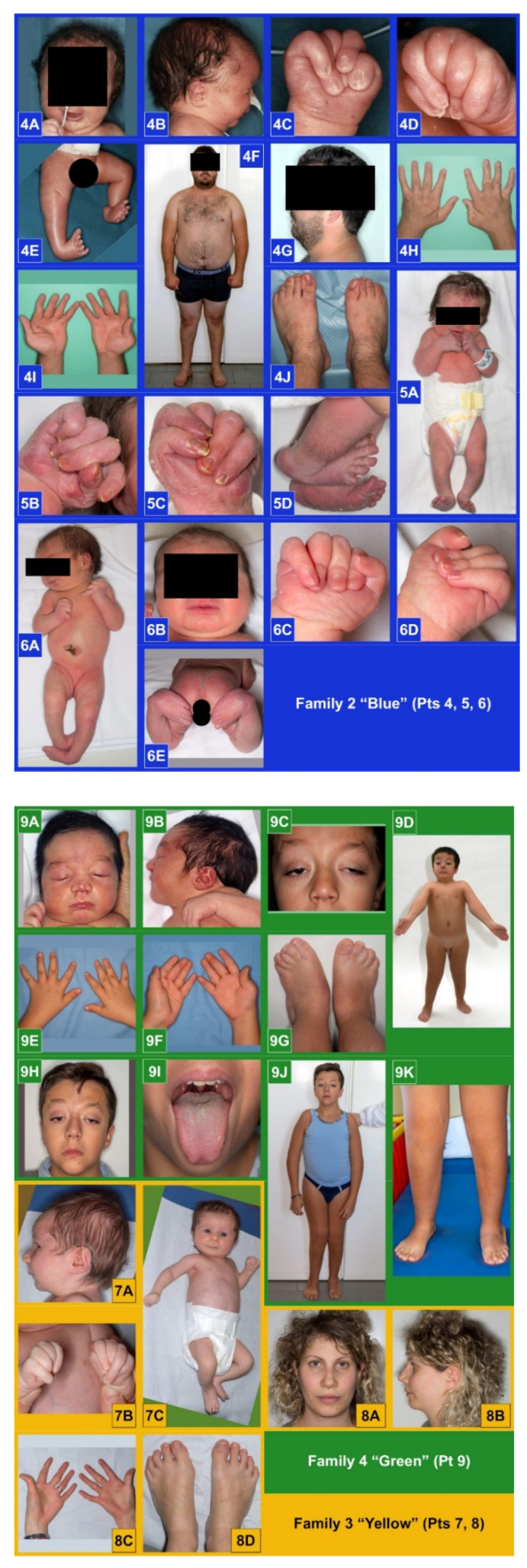

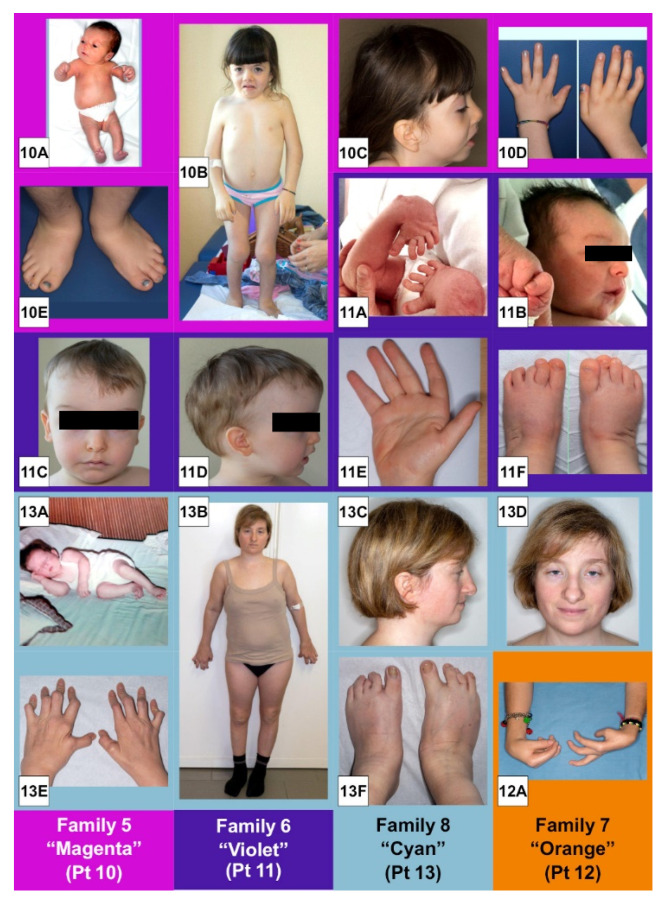
Phenotypic characteristics of the participants. Family 1 (Red background: **1A**–**E** pt 1; **2A**–**E** pt 2; **3A**–**C** pt 3). (**1A**) At birth. (**1B**–**E**) At 3 years and 8 months of age. (**2A**) In the neonatal period. (**2B**–**E**) At 24 years of age. (**3A**–**C**) At 48 years of age. Family 2 (Blue background: **4A**–**J** pt 4; **5A**–**D** pt 5; **6A**–**E** pt 6). (**4A**–**E**) At birth. (**4F**–**I**) At 26 years of age. (**5A**–**D**) At 1 month of life. (**6A**–**E**) At 1 month of life. Family 3 (Yellow background: **7A**–**C** pt 7; **8A**–**D** pt 8). (**7A**–**C**) At 1 month of life. (**8A**–**D**) At 27 years of age. Family 4 (Green backgorund: **9A**–**K** pt 9). (**9A,B**) In the neonatal period. (**9C**–**G**) At 7 years of age. (**9H**–**K**) At 12 years and 1 month. Family 5 (Magenta background: **10A**–**E** pt 10). (**10A**) At birth. (**10B**–**E**) At 4 years and 9 months of age. Family 6 (Violet background: **11A**–**F** pt 11). (**11A**,**B**) At birth. (**11C**–**F**) At 1 year and 7 months. Family 7 (Orange background: pt 12). (**12A**): Hands at 25 years of age. Family 8 (Cyan backgroun: **13A**–**F** pt 13). (**13A**) In childhood. (**13B**–**F**) At 35 years of age.

A total of 13 participants with arthrogryposis and otherwise normal neurological functions such as intellectual development and motor abilities, including amyoplasia, DA, and syndromic forms, were recruited for this study (Figure 1).

**Figure 2:**
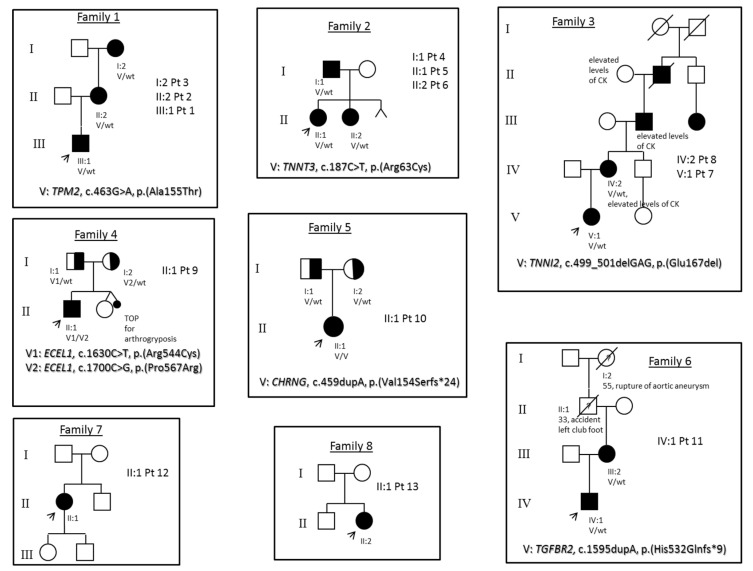
Pedigrees of the reported families. Pt = participants. V = variant. wt = wild-type. TOP = Termination Of Pregnancy. CK = creatine kinase. The arrow indicates the index case in each family.

All individuals showed involvement of all four extremities at birth. The group was composed of 9 females and 4 males. The age ranged from the prenatal period to 49 years. Family pedigrees are shown in Figure 2. None of the families reported consanguineous marriages, although consanguinity was suspected at least in Family 5, since the proband had a rare pathogenic variant in homozygosity. 

Table 1 summarizes the clinical features, instrumental findings, genetic analyses and performed treatments in our cohort. These elements are more widely described in Text S1 and Table S2. Clinical features are described in Supplementary Materials according to human phenotype ontology (HPO) terminology [29].

### 3.2. Clinical and Instrumental Findings in the Cohort

Prenatal ultrasound revealed abnormalities after the 20th week of gestation in 7 out of 12 cases (58%; examination was not performed on pt 3). In 3 familial cases, clinical prenatal findings of arthrogryposis were noticed (joint contractures of hands, ulnar deviation of hands, rocker bottom feet/talipes equinovarus) since the second trimester (Table S2). 

In the perinatal period, clinical findings were similar among the participants: most displayed ulnar deviation of the hand, overlapping fingers and/or toes, and camptodactyly. Only pt 12 had a distinct phenotype, with the typical presentation of amyoplasia: internal rotation of the shoulders, extended elbow, flexed wrist, camptodactyly, and equinovarus positioning of feet. 

In the postnatal period, 11/13 participants (85%) showed microretrognathia, which is reported as the most striking craniofacial effect of arthrogryposis [30,31]. Trismus was present in 8/13 participants (61%), usually determined by a variable association of microretrognathia and narrow mouth. It was observed not only in DA, as previously reported, but also in syndromic cases. Pterygium, which was previously reported in MPS and DA5D, was also present in three participants with dominant forms of DA, in the participant with amyoplasia and in the patient without a molecular diagnosis, mostly affecting thumbs or other fingers. Participants with a more complex phenotype achieved autonomous walking at the upper limit of physiological age (pt 5, pt 13) or with a delay (pt 7). No intellectual disability was present in our cohort, although speech delay was significant in pt 13, and less severe in pt 5. 

### 3.3. Molecular Diagnosis

Three families (eight participants) displayed a pathogenic variant in one of the genes encoding contractile proteins of the skeletal muscles (*TNNT3, TPM2, TNNI2*). Alterations in these genes can be responsible for either DA type 1 (DA1) or type 2B (DA2B), which are currently considered a clinical continuum rather than two separate entities. In family 2 bearing a *TNNT3* variant and diagnosed with DA2B, all affected individuals presented with trismus, previously described only in DA1. As a result, feeding difficulties were reported for these participants, especially in the neonatal period, and one patient needed speech therapy (pt 5). Trismus was present also in the three participants with a variant in the *TPM2* gene (pts 1, 2, 3), with a phenotype more compatible with DA1. They also showed hyperCKemia, serious in the mother, who had a recent history of muscular asthenia and paresthesias to the upper limbs, milder in the grandmother, and transient in the son. Variants in one of the two tropomyosin genes, *TPM2* and *TPM3*, have been associated with a number of different myopathies [32]. Following multidisciplinary care soon after birth, this group of participants in the DA1/DA2B spectrum had the best prognosis [14]. 

Pt 9 and pt 10 had a more complex phenotype, and their molecular diagnosis was difficult to reach. The evolution of the phenotype through the years was crucial to guide the diagnosis: DA5D in pt 9, with billelic variants in *ECEL1* that met the ACMG criteria of pathogenicity, and Escobar syndrome in pt 10, with a known pathogenic homozygous variant in *CHRNG* [28]. These two cases required three different molecular approaches, the highest number of analyses in the DA group. 

Pt 13 also had a complex syndromic phenotype, and despite multiple genetic tests, a molecular diagnosis could not be reached. She showed progressive camptodactyly and scoliosis, congenital cardiopathy, with right pulmonary artery hypoplasia, speech delay, and ocular findings. Her most debilitating aspect was progressive scoliosis, which did not improve despite repeated surgical treatments. Pt 12, who was diagnosed with amyoplasia, also had a complex phenotype and required five surgical interventions in order to improve his quality of life.

An important differential diagnosis was represented by LDS in pt 11. At birth, his clinical features were indistinguishable from those of DA, until a required echocardiography revealed bicuspid aortic valve and mild dilatation of the aortic root. These important elements, together with his family history, led to the correct diagnosis. 

## 4. Discussion

Molecular diagnosis of arthrogryposis remains challenging given the high genetic heterogeneity of conditions with this clinical sign.

Nowadays, improvements in diagnostic sensitivity of sonographic equipment and an increased alertness of gynecologists allow a better identification of elements suggestive of arthrogryposis in the prenatal period, especially in family cases, in which more targeted ultrasound scans are performed. Abnormalities in fetal movements and structural fetal anomalies can be found in some cases. Prenatal findings include polyhydramnios, craniofacial dysmorphisms (retrognathia), lung hypoplasia, pterygium, and intrauterine growth restriction (IUGR) [33,34]. Hydrops fetalis, nuchal edema, scoliosis, and absent stomach filling (due to lack of ability to swallow) were associated with unfavorable outcomes, implicating a neuromuscular etiology [35]. In our experience, if positioning of the fetus is favorable, careful examination of the ultrasound can also reveal hand clenching, adducted thumb, ulnar deviation of the hand, overlapping fingers, and rocker bottom foot/club foot, signs which are particularly indicative of arthrogryposis. 

A limitation of prenatal ultrasound is the inability to highlight anomalies at an early gestational age. It is reported that only about 25% of arthrogryposis cases can be detected within the 24th week, rarely influencing the couple’s decisions [36]. The management of a subsequent pregnancy is easier in the case of molecular diagnosis in a previous child. Molecular diagnosis would be useful in alerting physicians to plan the appropriate follow-up investigations for such cases [37,38]. While, in a previous article, absent stomach filling was associated with unfavorable outcome, implicating a neuromuscular etiology [35], in our cohort pt 5 showed reduced stomach bubble and presented a good prognosis. In our cohort, clinical findings in the perinatal period were similar among participants with different molecular diagnosis, and only post-natal follow-up with instrumental evaluations could help discriminate the most complex cases. In pt 11, LDS was diagnosed only after echocardiography had shown dilatation of the aortic root. The same variant was present in a peripheral blood sample from his mother, whose story was negative for arthrogryposis. This suggests a high degree of intrafamilial variability, but no further evaluations were performed to exclude a mosaicism. In a cohort of 65 LDS patients, finger contractures and talipes equinovarus were reported in 13% and 23% individuals, respectively [39]. Specific limb alterations compatible with arthrogryposis were observed in at least three further LDS patients, two of them bearing a variant in the *TGFBR2* gene [40,41,42]. So, we also advise considering this syndrome in the differential diagnosis of arthrogryposis. 

Some features present in the perinatal period showed an improvement during time due in particular to physiatric treatment. Early multidisciplinary care soon after birth [14] was successful in improving the arthrogryposis phenotype in all our participants, except the most severely affected, i.e., pt 13. The group of participants in the DA1/DA2B spectrum and the patient with LDS had the best prognosis, and in general, except for pt 4, required less surgical procedures than the case with DA5D (pt 9), one of the two syndromic cases (pt 13) and the case with amyoplasia (pt 12).

Molecular diagnosis allowed us to point out a significant intrafamilial variability among the individuals with DA. In the family with a *TPM2* variant described here, involvement of the extremities appeared to be severe in pt 1, moderate in the mother (pt 2), and milder in the grandmother (pt 3). Nevertheless, the generalized neuromuscular involvement was more serious in the mother, with hyperCKemia, muscular asthenia and paresthesias of the upper limbs, milder or transient in the grandmother (a singular elevated level of plasmatic CK) and transient in the son (elevated levels of plasmatic CK only in the past). Variants in the *TPM2* gene have been associated with a number of different myopathies. Most are heterozygous changes associated with an autosomal dominant disease [32]. Recently, the second case of recessively inherited TPM2-related Escobar variant of MPS and congenital myopathy in a patient from a consanguineous family was described [43]. In conclusion, we cannot exclude that these familial findings could be related to the role of the *TPM2* gene. A degree of intrafamilial variability is also described for the same *CHRNG* variants. In particular, the homozygous variant in the *CHRNG* gene we detected in pt 10 has already been reported in association with both the lethal and the non-lethal form of MPS. Although these different forms usually manifest in different families, it has been estimated that there is a 5% chance that a subsequent sibling will have a MPS subtype different than the proband, though concordance decreases for more distant relatives [44]. In addition, *CHRNG* variants and variants in other components of the embryonal acetylcholine receptor may present with fetal akinesia deformation sequence (FADS) without pterygia. These considerations were important for genetic counselling of the family. 

Molecular diagnosis was also important for the follow-up. Due to possible respiratory distress in patients with *ECEL1* variants, for pt 9, we recommended not only orthopedic, but also respiratory follow-up [45,46]. In the participant with LDS, we planned not only skeletal investigations, but also vascular, ocular, and cardiological assessments. In pt 10, scoliosis and an already described anomaly of the craniovertebral junction (fusion of cervical vertebrae and clefting of vertebral bodies) were discovered during skeletal survey [4]. Follow-up at later ages was not sufficient to reach a molecular diagnosis in pt 13, despite WES analysis and several cytogenetic investigations. We cannot exclude the possibility of multiple loci contributing to the phenotype with an oligogenic mode of inheritance, as recently described [27].

## 5. Conclusions

We have described a series of participants affected by arthrogryposis, complete with detailed clinical and molecular information that may help to portray the natural history of this phenotype. 

During the prenatal and perinatal periods, clinical features are sometimes similar among different conditions, but over time, specific elements of a particular disease tend to appear. Clinical examination of the parents is helpful to suspect a specific condition and to define the recurrence risk. Since distal arthrogryposis (DA) has a better prognosis than other forms, molecular analysis can help reassure the parents of a newborn. In the same way, a molecular confirmation can prepare parents for conditions with a worse prognosis. Due to a varied differential diagnosis, early genetic testing can also help to avoid unnecessary investigations in children. Molecular diagnosis also helps in the planning of a specific follow-up. Finally, due to a considerable genetic heterogeneity, we confirm the precious contribution of next generation sequencing (NGS) in the molecular diagnosis of arthrogryposis.

## Figures and Tables

**Table 1:** Summary of the clinical features, instrumental findings, genetic analyses and performed treatments in our cohort. Each column corresponds to a patient. Pt = patient. Fy = family. M = male. F = female. − = absent. + = present. PP = prenatal period. Y = years. D = days. m = months. N.A. = not avalaible. Het = heterozygous. hom = homozygous. comp = compound. DA = distal arthrogryposis. EVMPS = nonlethal Escobar variant of multiple pterygium syndrome. LDS = Loeys-Dietz syndrome. A = amyoplasia. U = undefined.

	Pt 1 (Fy1)	Pt 2 (Fy1)	Pt 3 (Fy1)	Pt 4 (Fy2)	Pt 5 (Fy2)	Pt 6 (Fy2)	Pt 7 (Fy3)	Pt 8 (Fy3)	Pt 9 (Fy4)	Pt 10 (Fy5)	Pt 11 (Fy6)	Pt 12 (Fy7)	Pt 13 (Fy8)	Total
**General information**														
Sex	M	F	F	M	F	F	F	F	M	F	M	F	F	
Age at first evaluation	PP	20 y	44 y	1 d	PP	PP	1 m	27 y	6 d	1 d	1 y	3 y	16 y	
Current age (years)	4	25	49	31	5	4	2	29	15	9	2	31	37	
**Prenatal findings**														
Ultrasound abnormalities	+	−	N.A.	+	+	+	+	−	−	+	−	+	−	7/12 = 58%
**Postnatal features**														
Short stature	−	+	−	+	−	+	−	−	+	+	−	−	+	6/13 = 46%
Ptosis	−	−	−	−	−	−	−	−	+	+	−	−	+	3/13 = 23%
Epicanthus	−	−	−	−	−	−	−	−	+	+	−	−	+	3/13 = 23%
Narrow mouth	+	+	−	+	+	+	−	−	−	+	−	−	+	7/13 = 54%
Microretrognathia	+	−	−	+	+	+	+	+	+	+	+	+	+	11/13 = 85%
Trismus	+	+	+	+	+	+	−	−	−	+	−	−	+	8/13= 61%
Myopathic facies	−	−	−	−	−	−	−	−	+	+	−	−	+	3/13= 23%
Ulnar deviation of the hand	+	+	−	+	+	+	+	+	+	+	+	−	+	11/13 = 85%
Hand clenching	+	−	−	+	+	+	+	−	+	+	+	−	−	8/13 = 61%
Adducted thumb	+	+	−	+	+	+	+	+	+	+	+	−	+	11/13 = 85%
Overlapping fingers	+	−	−	+	+	+	+	+	−	+	+	+	−	9/13 = 69%
Camptodactyly	+	−	−	+	+	+	−	+	+	+	−	−	+	8/13 = 61%
Single transverse palmar crease	+	−	−	+	+	+	−	−	+	+	+	−	+	8/13 = 61%
Talipes equinovarus	+	+	+	+	−	+	−	−	−	−	+	+	+	8/13 = 61%
Rocker bottom foot	−	−	−	−	+	−	+	+	+	+	−	−	−	5/13 = 38%
Overlapping toes	+	+	+	+	+	−	+	−	+	−	+	+	+	10/13 = 77%
Prominent calcaneus	+	−	−	+	+	−	+	+	+	+	+	+	−	9/13 = 69%
Pterygium	−	+	+	+	−	−	−	−	+	+	−	+	+	7/13 = 54%
Scoliosis	−	−	+	−	−	−	−	−	+	+	−	+	+	5/13 = 38%
Hip dysplasia	−	−	−	+	−	−	−	−	+	+	−	−	−	3/13 = 23%
Other clinical findings	+	+	+	−	+	+	+	+	+	+	+	+	+	12/13 = 92%
**Genetic testing**														
Molecular diagnosis	het NM_003289.4(TPM2): c.463G>A, p.(A155T)			het NM_006757.3(TNNT3): c.187C>T, p.(R63C)			het NM_001145829.1(TNNI2): c.499_501del, p.(E167del)		comp het NM_004826(ECEL1): c.[1630C>T];[1700C>G], p.[(R544C)];[(P567R)]	hom NM_005199.4(CHRNG): c.459dup, p.(V154Sfs*24)	het NM_ 001024847.2(TGFBR2): c.1595dupA, p.(H532Qfs*9)	/	/	11/12 = 92%
Diagnosis	DA 1			DA 1			DA 2B		DA 5D	EVMPS	LDS 2	A	U	
**Treatment**														
Physiotherapy	+	−	−	−	+	+	+	−	+	+	+	−	+	8/13 = 61%
Orthopedic devices	+	+	−	+	+	+	+	+	+	+	+	+	+	12/13 = 92%
Number of surgeries	4	2	1	5	1	−	1	1	5	2	2	5	6	12/13 = 92%

## Data Availability

The data that support the findings of this study are available from the corresponding author upon reasonable request.

## Supplementary Materials

The following are available online at https://www.mdpi.com/article/10.3390/genes13010029/s1. Text S1: Medical history, clinical features, instrumental findings, genetic analyses and performed treatments of the described participants. Table S1: Classification of variants associated with arthrogryposis in our cohort, according to the recommendations of the American College of Medical Genetics (ACMG) [28]. Table S2: Clinical features, instrumental findings, genetic analyses and performed treatments of the described participants. Each column corresponds to a patient. The feature has to be considered bilateral, if not otherwise specified. Pt = patient. Fy = family. M = male. F = female. − = absent. + = present. N.A. = not avalaible. het = heterozygous. hom = homozygous. comp = compound. NGS = next generation sequencing. NIPT = non-invasive prenatal testing. CGH-array = comparative genomic hybridization array. WES = whole exome sequencing. FISH = fluorescent in situ hybridization.
