# Supplementary material for: Clinical and Genetic Findings in a Series of Eight Families with Arthrogryposis
Source: Genes (Basel). 2021 Dec 23;13(1):29. doi: 10.3390/genes13010029 (PMC8774604; doi:10.3390/genes13010029)
Supplement: Supplementary file 1 [file genes-13-00029-s001.zip › genes-1508372-Sup update/Table S1.pdf]

Table S1: Classification of variants associated with arthrogryposis in our cohort, according to the recommendations of the American College of Medical Genetics (ACMG) [28].

Family (Patients #)	Gene	Variant	Classification (class)	ACMG Criteria
Fy1 (1-3)	*TPM2*	NM_003289.4:c.[463G>A];[=] NP_003280.2:p.[(A155T)];[=]	likely pathogenic (4)	PM2, PM5, PP1, PP2, PP3, PP5
Fy2 (4-6)	*TNNT3*	NM_006757.3:c.[187C>T];[=] NP_006748.1:p.[(R63C)];[=]	likely pathogenic (4)	PM5, PM2, PP1, PP3, PP5 (used as moderate)
Fy3 (7-8)	*TNNI2*	NM_001145829.2:c.[499_501del];[=] NP_001139301.1:p.[(E167del)];[=]	likely pathogenic (4)	PM1, PM2, PM4, PP1, PP3, PP5
Fy4 (9)	*ECEL1*	NM_004826.4:c.[1630C>T];[1700C>G] NP_004817.2:p.[(R544C)];[(P567R)]	likely pathogenic (4)	PM2, PM3, PP3, PP5, PP4
Fy5 (10)	*CHRNG*	NM_005199.4:c.[459dup];[459dup] NP_005190.4:p.[(V154Sfs*24)];[(V154Sfs*24)]	pathogenic (5)	PVS1, PM2, PM3, PP4, PP5
Fy6 (11)	*TGFBR2*	NM_001024847.2:c.[1595dup];[=] NP_001020018.1:p.[(H532Qfs*9)];[=]	likely pathogenic (4)	PM2, PM4, PP3, PP1

References

[28]. Richards, S.; Aziz, N.; Bale, S.; Bick, D.; Das, S.; Gastier-Foster, J.; Grody, W.W.; Hegde, M.; Lyon, E.; Spector, E.; et al. Standards and guidelines for the interpretation of sequence variants: A joint consensus recommendation of the American College of Medical Genetics and Genomics and the Association for Molecular Pathology. Genet. Med. 2015, 17, 405–424. https://doi.org/10.1038/gim.2015.30.
