# Supplementary material for: Clinical and Genetic Findings in a Series of Eight Families with Arthrogryposis
Source: Genes (Basel). 2021 Dec 23;13(1):29. doi: 10.3390/genes13010029 (PMC8774604; doi:10.3390/genes13010029)
Supplement: Supplementary file 1 [file genes-13-00029-s001.zip › genes-1508372-Sup update/Text S1.pdf]

Text S1: Medical history, clinical features, instrumental findings, genetic analyses and performed treatments of the described participants.

Family 1.

<u>Pt 1</u> is the son of pt 2 and the grandson of pt 3. He is the only child of a woman of Italian origins and a man from the Dominican Republic. He was followed from the prenatal period, because prenatal ultrasounds from 20th week of gestation found bilateral hand clenching with overlapping fingers and left rocker bottom foot. Karyotype on amniotic fluid resulted normal (46,XY). He was born at 40 weeks by urgent caesarean section due to dynamic dystocia. Birth parameters were: weight 3.124 kg (10-25th percentile), length 48 cm (10th percentile), head circumference 35 cm (50th percentile). Apgar scores were 7, 10 at 1 and 5 minutes respectively. He was first evaluated at 2 days of life when we noticed: microretrognathia, hand clenching, bilateral ulnar deviation of the hand, adducted thumb, joint contracture of the 3rd finger, overlapping fingers (F13, F21, F23 and F43 on right side; F21, F23 and F43 on left side), left talipes equinovarus, right overlapping toes (F21). He also showed: trismus, cyanosis, irregular breathing, hypertonia and sucking difficulty. Head control was acquired at 2 months, walking with support at 12 months, walking alone at 14 months, babbling at 7 months, speech at 12 months. He was treated with physiotherapy during the first 2 years. Bilateral clubfoot was treated according to Ponseti method: serial manipulation and casting, left Achilles tendon tenotomy at 2 months, then foot abduction orthosis and surgery at 2 years and 11 months for treatment of left foot. Since the age of 12 months until now, that he is 4 years and 10 months, he has been using orthopedic shoes and orthotics during the day, and a foot abduction orthosis during the night, to prevent equinovarus relapse. Moreover, upper limb splints have been used in the nighttime since the age of 5 months. He is waiting for surgery to correct adducted thumb and ulnar deviation. At 2 years he underwent surgery for left inguinal hernia and frenulotomy of the tongue. Cerebral MRI found mild hypoplasia of the lower portion of the cerebellar vermis and big cisterna magna. AOEs, hip ultrasound, abdominal ultrasound, echocardiography, X-ray of the hand and electroneurography/electromyography (ENG/EMG) were normal. In 2 episodes he had elevated level of creatine kinase (CK) in blood (831 U/l, 377 U/l), that later normalized. Due to clinical findings and family history, the diagnostic hypothesis was of DA. At the last clinical evaluation, at 3 years and 10 months, we pointed out: height 96 cm (10th-25th percentile), weight 13 kg (3-10th percentile), head circumference 49 cm (10th-25th percentile), thick helix, microretrognathia, small mouth with trismus, chin with horizontal crease, ulnar deviation of the hand, adducted thumb, bilateral single transverse palmar creases, pes planus, prominent heel bone (> on right side), overlapping toes (F21 and F23 bilaterally).

<u>Pt 2</u> is a 25-year-old woman of Italian origins. She was first evaluated at 20 years, during her pregnancy (of pt 1). No abnormalities are reported in the prenatal period. At birth she showed: bilateral clubfoot, ulnar deviation of the hand, adducted thumb, right cutaneous syndactyly I-II fingers and convergent strabismus of left eye. She reached normal psychomotor development. She underwent surgery for both feet and right hand around 6 months. She used orthopedic shoes during childhood and she is now using orthotics. This year she underwent eye surgery for strabismus. She has recently reported muscular asthenia and paresthesias on right upper limb. Blood tests found constant elevated levels of CK (677-1606 U/l). At the last clinical evaluation, at 25 years,

she showed: short stature (height 146.5 cm, <3rd percentile), decreased body weight (40 kg, <3rd percentile), head circumference 52.5 cm (3rd-10th percentile), small mouth with trismus, bilateral pterygia of thumb (more pronounced on right side), flexion stiffness of the metacarpophalangeal joint of the right thumb, overlapping toes (F21 and F23 on left side; F12 on right side), slight difficulty in flexing the toes.

Pt 3 is a 49-year-old woman of Italian origins. She was first evaluated at 44 years, due to family history. No information or instrumental evaluation is available in the prenatal period. At birth, she showed bilateral clubfoot, which required surgery at around 6 months. She reached normal psychomotor development. Recently, a blood test found elevated levels of CK (200 U/l) with no further analysis available. She denies muscular problems. At the last clinical evaluation, at 44 years, she showed: height 152 cm (3rd-10th percentile), trismus, scoliosis, bilateral pterygia of thumb (more pronounced on left side), clinodactyly of the 2nd left finger, inability to flex the toes.

Genetic testing. An NGS panel of 9 genes associated with DA (*CHST14, FBN2, MYBPC1, MYH3, MYH8, PIEZO2, TNNI2, TNNT3, TPM2*), performed when pt 1 was 6 months old, revealed the heterozygous missense variant c.463G>A, p.(Ala155Thr) in the *TPM2* gene (NM_213674.1) in pt 1. Variants in the *TPM2* gene are responsible for DA1, DA2B, CAP myopathy 2 and Nemaline myopathy 4. This variant is located at a highly conserved position and is predicted in silico to be deleterious [1-4]. It is not reported in the gnomAD v2.1.1 database, but it has already been described in patients with arthrogryposis or unspecified congenital myopathies [5-8]. Direct sequencing confirmed the presence of this variant in pt 2 and pt 3 as well. Since it segregated with the disease in family 1, it was classified as likely pathogenic.

Family 2.

Pt 4, male, is the second child of a healthy, non-consanguineous couple of Roma ethnic origin. The older brother, born preterm, died at 2 days of life due to cerebral hemorrhage. The younger sister is healthy. During pregnancy polyhydramnios was noticed. He was born by C-section at the 37th week of gestation with acute fetal distress. Birth parameters were: weight 2.270 kg (3rd-10th percentile), length 44 cm (<3rd percentile), head circumference 34 cm (50-75th percentile). Apgar scores were 2, 7, 8 at 1, 5 and 10 minutes respectively. He required aspiration and ventilation with ambu and oxygen. He showed hand clenching, bilateral adducted thumb, overlapping fingers, camptodactyly of the 3rd and of the 4th toe, single transverse palmar crease, bilateral talipes equinovarus, congenital dysplasia of right hip, mild limb undergrowth, short palpebral fissures, long philtrum, microretrognathia and low-set ears. He was kept in the incubator for about 2 weeks and fed by nasogastric tube. Neonatal abdominal ultrasound revealed a mild ectasia of the calico-pyelic cavity. During follow-up, no psychomotor delay was reported. He was treated with casts at the lower limbs and he underwent 5 surgical procedures for the treatment of extremities: feet at 1 year, left hand at 11 years, right foot at 13 years (twice) and left foot at 14 years. He is still using orthopedic shoes. Foot X-ray demonstrated bone dysmorphisms compatible with the clinical signs, without osteolytic or osteoblastic changes. Echocardiography, cerebral ultrasound and electroencephalogram (EEG) were normal. The diagnostic hypothesis was of DA1/DA2B. At the last clinical evaluation, at 31 years, he showed short stature (height 154.5 cm, <3rd percentile), weight 86 kg (>97th percentile), head circumference 58 cm (>97th percentile, mean/+2SD), flat face,

low-set ears, ulnar deviation of the hands, bilateral pterygia of thumb, camptodactyly of fingers (> right hand), bilateral single transverse palmar crease, hypoplasia of calf muscles, bilateral overlapping toes, F12, prominent calcaneus, a single hypopigmented irregular dorsal skin patch of 4 cm, trismus.

Pt 5 is the first daughter of pt 4. Prenatal ultrasound at 20th week of gestation found left hand clenching, ulnar deviation of the right hand, rocker bottom foot, reduced stomach bubble. She was born at 41 weeks by natural delivery with amniotic fluid dyed by meconium. Parameters were: weight 2.745 kg (3rd-10th percentile), length 48 cm (10-25th percentile), head circumference 35 cm (50-75th percentile). Apgar scores were 8, 9 at 1 and 5 minutes respectively. Evaluation at 2 days of life showed microretrognathia, short neck with a hint of pterygium, ears with overfolded helix, hand clenching, bilateral ulnar deviation of the hand, bilateral overlapping fingers (F23, F43 and F53), bilateral rocker bottom foot and prominent calcaneus. Due to incomplete sucking she was fed by nasogastric tube until the 23rd day of life. No motor delay was reported. Since birth, she has been treated with serial static splints for upper and lower limbs, and with physiotherapy until 3 years of age. Then, she had been using foot orthoses since she had started walking, at 1 year and 4 months, until 2 years and 9 months. In the same period she also stopped upper limb splints. At present, at 5 years and 8 months, she is waiting for surgery due to joint contracture of the left 3rd finger and she is in follow-up for speech delay. Acoustic otoemissions (AOEs), fundus examination, abdominal ultrasound, hip ultrasound, cerebral ultrasound, X-ray of foot, hands and spine were normal. Echocardiography at 1 year found mild dilatation of ascendant aorta, to be controlled. Due to clinical findings and family history, the diagnostic hypothesis was of DA. At the last clinical evaluation, at 1 year and 2 months, she showed: length 67.5 cm (10-25th percentile), weight 7.325 kg (10-25th percentile), head circumference 43.5 cm (10th percentile), nevus flammeus of the forehead, mild microretrognathia, ogival palate, short neck, ears with thickened helices and squared superior portion of helix (>right side), slight stiffness in passive extension of the lower limbs, ulnar deviations of fingers (>left side), flat feet with prominent calcaneus. The left hand showed: hand clenching, adducted thumb, overlapping fingers (F23, F24), joint contracture of the 3rd finger and single transverse palmar crease.

Pt 6 is the younger sister of pt 5 who was also monitored after the prenatal period, because prenatal ultrasounds since 20th weeks of gestation found bilateral hand clenching, adducted thumb, rocker bottom foot, choroid plexus cyst and decreased fetal movement. The parents decided not have invasive prenatal diagnosis carried out. She was born at 39 weeks by caesarean section due to breech presentation. Birth parameters were: weight 2.554 kg (3rd-10th percentile), length 42 cm (<3rd percentile), head circumference 34 cm (25th-50th percentile). Apgar scores were 9, 10 at 1 and 5 minutes respectively. She was first evaluated at 2 days of life. We noticed: microretrognathia, hand clenching, bilateral ulnar deviation of the hand, adducted thumb, overlapping fingers (F12 and F54 on right side; F12, F13 and F32 on left side), talipes equinovarus. She was treated with physiotherapy until 3 years and serial static splints for upper limbs, since 1 month. Bilateral clubfoot was treated according to Ponseti method (serial manipulation and casting, followed by the use of a foot abduction orthosis). Now that she is 4 years and 9 months old, parents report that she does not need therapy and that psychomotor development is normal. Due to laryngeal stridor she carried out investigations with the exclusion of laryngomalacia. AOEs, hip ultrasound, cerebral ultrasound and echocardiography were normal.

Genetic testing. An NGS panel of 9 genes associated with DA (*CHST14, FBN2, MYBPC1, MYH3, MYH8, PIEZO2, TNNI2, TNNT3, TPM2*), performed after birth of pt 5, revealed the heterozygous missense variant c.187C>T, p.(Arg63Cys) in the *TNNT3* gene (NM_ 006757.3) in pt 4 and pt 5. This variant has already been described in association with DA1 and DA2B [9,10]. The presence of this variant was subsequently confirmed by direct sequencing in pt 6 as well and was therefore considered likely pathogenic. Previous blood standard karyotype in pt 4 was normal (46,XY).

Family 3.

Pt 7 is a female, the only child of a non-consanguineous couple of Italian origins. The mother is pt 8. Different relatives are reported to have anomalies in the extremities compatible with DA: mother, maternal grandfather, the father and a female first-degree cousin of this last relative. There is a clinical variability of clinical features: only the hands are involved in the maternal grandfather and in his cousin; both hands and feet in the great grandfather of pt 9 (see Figure 2). No genetic testing had been initiated prior to our genetic counseling. Family tree was also positive for elevated levels of CK in the maternal line (mother, maternal grandfather and his mother). She was evaluated for the first time at 1 month. Pregnancy was characterized by oligohydramnios and decreased fetal movement. She was born at 41 weeks by induced vaginal delivery. Birth parameters were: weight 2.430 kg (<3rd percentile), length 47 cm (3rd-10th percentile), head circumference 33 cm (10th-25th percentile). Apgar scores were 9, 10 and 10 at 1, 5 and 10 minutes respectively. We noticed at birth: hand clenching, ulnar deviation of the hand, bilateral adducted thumb, overlapping fingers (F23 and F54 on right side; F21, F23, F43 and F45 on left side), bilateral rocker bottom foot and prominent calcaneus. Since 2 days of life she experienced episodes of dark vomit and polypnea, later solved in the first week of life. She was treated with orthoses at the hands. Currently she is waiting for surgery at the thumbs and she is still treated with physiotherapy at the hands and orthopedic shoes with orthotics. She acquired head control at 6 months, walking with support at 14 months, walking alone at 16 months, babbling at 14 months, speech at 18 months. The following evaluations were normal: AOEs, fundus examination, echocardiography, hip ultrasound, abdominal ultrasound, cerebral ultrasound. At the last clinical evaluation, at 7 months, she showed: length 64 cm (10th percentile), weight 6.725 kg (5th-10th percentile), head circumference 43.5 cm (75th-90th percentile), brachycephaly, microretrognathia, hand clenching, ulnar deviation of the hand, bilateral adducted thumb, overlapping fingers (F23 and F54 on right side; F21, F23, F43 and F45 on left side), bilateral rocker bottom foot, prominent calcaneus and overlapping toes (F21 bilaterally).

Pt 8 is the mother of pt 7. She is the first child of a non-consanguineous couple of Italian origins. The younger brother is healthy. We could examine her at 27 years of age, during the genetic counseling for the daughter (pt 7). Pregnancy was unremarkable. She was born at term by caesarean section due to previous caesarean section. Birth parameters were: weight 3180 kg (25th-50th percentile), length 50 cm (50th percentile). No additional data are available. At birth she showed: arachnodactyly, camptodactyly, bilateral adducted thumb, ulnar deviation of the hand, bilateral rocker bottom foot, prominent calcaneus, mild flexion contractures of the knees and of the elbows. She also showed: microretrognathia. At 22 years, a right ovarian dermoid cyst was revealed. She reached normal psychomotor development. She was treated with serial static splints at the hands since 9 months until 5 years and 6 months and she

underwent 1 surgical procedure at the right thumb at 5 years. She was also treated with orthoses at the hands from 9 months until 5 years and 6 months. The following evaluations were normal: AOEs, ECG, echocardiography, abdominal ultrasound, X-rays of both hands, feet, forearms and lumbosacral spine. At the clinical evaluation, at 27 years, she showed: height 161 cm (25th-50th percentile), weight 67 kg (75th-90th percentile), head circumference 54.5 cm (50th-75th percentile), ulnar deviation of the hands, hyperextension of the distal phalanx of the thumb, contracture of the distal interphalangeal joint of the 4th toe and extension of the proximal ones, pes planus. She also showed mild microretrognathia.

Genetic testing. An NGS panel of 11 genes associated with DA (*CHST14, ERBB3, FBN2, MYBPC1, MYH3, MYH8, PIEZO2, RAPSN, TNNI2, TNNT3, TPM2*), performed after the first evaluation of the family, revealed the heterozygous deletion variant c.499_501delGAG, p.(Glu167del) in the *TNNI2* gene (NM_ 001145829.1) in pt 7. This variant has already been described in association with DA2B in a family [13] and in association with DA1 in another family [10] and was therefore considered likely pathogenic. The presence of this variant was subsequently confirmed by direct sequencing in pt 8 as well.

Family 4.

Pt 9 is the first child conceived via intracytoplasmic sperm injection (ICSI) of a non-consanguineous couple of Italian origins. His younger sister is healthy and she was born after a twin pregnancy in which features compatible with arthrogryposis were discovered in the other twin, so that selective termination has been requested. He was evaluated for the first time at 6 days of life, then every successive year. During pregnancy decreased fetal movement was noticed. Karyotype on amniotic fluid resulted normal (46,XY). He was born at 39 weeks by vaginal delivery. Birth parameters were: weight 3.425 kg (50th-75th percentile), length 51 cm (75th percentile), head circumference 37 cm (90th-97th percentile). Apgar scores were 9 and 10 at 1 and 5 minutes respectively. At birth he showed: hand clenching, right camptodactyly of the 3rd finger, slight ulnar deviation of the hand, right adducted thumb, bilateral rocker bottom foot, left genu recurvatum, limited elbow and right knee extension. Other features were: flat face, hypertelorism, high palate, short neck, hyperextended head, pilonidal sinus and left cryptorchidism. Phenotype changed significantly at 10 years and 6 months, when multiple pterygia were noticed and Escobar syndrome was suspected. Physiotherapy was performed regularly in the first 3 years, then mostly following surgical events. He also practiced swimming. He used serial static splints and then an orthosis for the right hand until 10 months. A nighttime static splint was adopted also to reduce left genu recurvatum in the first three years. Foot orthoses were used until 13 months to correct the bilateral rocker bottom foot, and ankle-foot orthoses were initially worn to facilitate standing. After 20 months orthotics and orthopedic shoes were introduced. A customized sitting system was used from 7 to 15 months, to improve trunk alignment. Scoliosis was noticed at 6 years and an orthopedic corset was introduced at 12 years until surgery. He underwent 5 surgical procedures: right hip reconstructive surgery at 6 years; orthopedic surgery to correct left rocker bottom foot at 9 years; distal right femoral epiphysiodesis at 11 years and proximal right tibial epiphysiodesis at 12 years, to reduce valgus knee; vertebral arthrodesis at 14 years. After the last surgery he stopped using orthopedic shoes and orthotics. At 3 and 4 years he underwent surgery for left cryptorchidism. Head control was acquired after 4 months, sitting with upper limb support at 7 months, standing with support at

14 months, walking alone at 20 months, babbling and speech at 12 months. Hip ultrasound found congenital dislocation at the right side and subluxation at the left side, first treated with retractor. Spinal X-ray showed dorsolumbar scoliosis. Pelvis X-Ray at 11 years showed bilateral Legg-Calve-Perthes disease. MRI of right knee found tibial femoral misalignment with retroposed femoral axis. Ophtalmological evaluation revealed Duane anomaly and astigmatism. The following evaluations resulted normal: AOEs, echocardiography, abdominal ultrasound, EEG, skull, hand and foot X-rays, dynamic cervical X-ray, cerebral and of the spinal cord MRI, ENG/EMG. At the last clinical evaluation, at 15 years and 3 months, he showed: short stature (height 155.5 cm, <3rd percentile), weight 58.5 kg (50th-75th percentile), head circumference 54.5 cm (50th-75th percentile), myopathic and flat facies, bilateral ptosis, bilateral epicanthus, highly arched eyebrow, sparse lateral eyebrow, Duane anomaly, high palate, furrowed tongue, mild microretrognathia, short neck, 3 irregular areas of hyperpigmentation on the trunk, pilonidal sinus, loss of gluteal subcutaneous adipose tissue on the right side, scoliosis, camptodactyly of left 2nd finger, single transverse palmar crease on the right hand, pes planus, overlapping toes (F21 and F23 on the left side; F23 on the right side). Pterygia were present in different areas: digits (2nd and 3rd left fingers), right thumb, neck, antecubital, popliteal. He also showed multiple grooves: gluteal on the right side, anterior tibialis (> on the right side). We noticed abnormal movements: limited pronosupination of the upper limb, difficulty lifting the upper limbs above the head, difficulty in bending of the knees, hyperextensible distal phalanx of right thumb.

<u>Genetic testing.</u> Whole Exome Sequencing (WES) revealed the compound heterozygous missense variants NM_004826:c.[1630C>T];[1700C>G], p.[(Arg544Cys)];[(Pro567Arg)] in the *ECEL1* gene. Both are extremely rare and never homozygous in the reference population database gnomAD v2.1.1, and are predicted in silico to have a deleterious effect on the protein product [5]. ClinVar reports p.(Arg544Cys) as homozygous in a patient with distal arthrogryposis, and p.(Pro567Arg) in an individual affected with an unspecified condition [12]. These variants were therefore interpreted as likely pathogenic. Previous molecular analysis (*FLNB* gene and NGS panel of 9 genes associated with DA, *CHST14, FBN2, MYBPC1, MYH3, MYH8, PIEZO2, TNNI2, TNNT3, TPM2,* plus *CHRNG* gene) resulted normal.

Family 5.

<u>Pt 10</u> is the only child of a non-consanguineous couple of Italian origins. She was evaluated for the first time at birth. During pregnancy polyhydramnios and short fetal femur length were noticed. Karyotype on amniotic fluid resulted normal (46,XX). She was born at 39 weeks by caesarean section due to irregular uterine contractions and deceleration of the heartbeat. Birth parameters were: weight 2.560 kg (3rd-10th percentile), length 44 cm (<3rd percentile), head circumference 34 cm (50th percentile). Apgar scores were 9 and 10 at 1 and 5 minutes respectively. She showed 2 episodes of cyanosis in the first hours of life and mild hyperbilirubinemia, so she underwent cardiorespiratory monitoring and phototherapy. At birth we noticed: hand clenching, slight camptodactyly (in particular distal inter-phalangeal of the 3rd finger bilaterally), ulnar deviation of the hand, bilateral adducted thumb, overlapping fingers (F12, F23, F43 and F54 on right side; F13, F21, F23 and F43 on left side), congenital bilateral talipes calcaneovalgus, flexion contractures (in particular knees; impossible extension). She also showed: prominent nasolabial fold, small mouth, microretrognathia, attached earlobe on the right side, short

and webbed neck, slight hypoplastic labia majora. Trunk control was reached at 10 months, standing with support at 16 months, walking with support at 18 months, alone at 20 months, babbling at 8 months and speech at 12 months. She reached normal psychomotor development, but due to mild coronal velopharyngeal insufficiency, she shows dyslalia and nasal speech. Physiotherapy started in the first month and it was attended regularly until 8 years. She was treated with serial static splints at the upper limbs until 14 months, at the lower limbs until 12 months. At 12 months knee-ankle-foot orthoses were introduced to facilitate standing with support and later to help her walking. At 5 years they were substituted by carbon-leaf ankle-foot orthoses, which were used until 8 years. She underwent 1 surgical procedure at 7 years, to correct rocker bottom feet and reduce knee flexion (bilateral femoral epiphysiodesis). She is waiting for bilateral proximal femural osteotomy to reduce hip flexion. Total body X-ray showed subluxation with ulnar deviation of the hands. Static and dynamic cervical X-ray showed initial anterior sliding of C2 over C3 during flexion and partial fusion of C3 and C4. Magnetic Resonance Imaging (MRI) of the spine showed anterior and posterior cleft of C1, fusion of the spinous processes of C2-C3, D3-D4 and D5-D9, smaller and lower vertebral bodies in cervicodorsal region and mild dorsal scoliosis. MRI of the brain showed mega cisterna magna. The following evaluations were normal: AOEs, fundus examination, ECG, echocardiography, hip ultrasound, abdominal ultrasound, EEG, MRI of the spinal cord and ENG/EMG. The first diagnostic hypothesis was of DA. Later, due to phenotypic changes since 4 years, Escobar syndrome was suspected. At the last clinical evaluation, at 9 years and 11 months, we observed: short stature (height 115.5 cm, <3rd percentile), low weight (20 kg, <3rd percentile), head circumference 50.5 cm (10th percentile), myopathic facies, bilateral ptosis, downslanted palpebral fissures, bilateral epicanthus, short nose, anteverted nares, mild prominent nasolabial fold, small open mouth, trismus, high palate, smooth philtrum, attached and hypoplastic earlobes, ulnar deviation of the hands, mild camptodactyly of fingers, bilateral clinodactyly of the 5th finger, talipes calcaneovalgus and 2-3 right toe cutaneous syndactyly. Pterygia were present in different areas: neck, axillae, antecubital, digits and popliteal area. She also showed: pectus carinatum, lumbar hyperlordosis, reduced neck extension, limited pronosupination of the upper limb, hypotrophy of calf muscles and calcaneal linear vertical creases.

Genetic testing. An NGS panel of 9 genes associated with DA (*CHST14, FBN2, MYBPC1, MYH3, MYH8, PIEZO2, TNNI2, TNNT3, TPM2)* plus *CHRNG* gene, revealed the homozygous frameshift variant c.459dupA, p.(Val154Serfs*24) in the *CHRNG* gene (NM_ 005199.4). Both parents are carriers. This variant has already been described in association with Escobar syndrome and was therefore considered pathogenic [14-17]. Previous genetic analysis (array-based Comparative Genomic Hybridization (aCGH) and molecular analysis of the *DMPK* gene) resulted normal.

Family 6.

Pt 11 is the only child of a non-consanguineous couple of Italian origins. Family history was positive for tall stature in both lineages. The maternal grandfather, who died at 33 years by accident, showed left clubfoot at birth. The maternal great-grandmother died at 55 years by rupture of aortic aneurysm (see Figure 2). The 37-year-old mother had postpartum hemorrhage and she is myopic (-3.25 diopters). She showed at a clinical evaluation: tall stature, blue sclerae, bifid uvula, bilateral pes planus and Beighton score 5/9. Patient 11 was

evaluated for the first time at 6 months. Prenatal ultrasounds were normal. Increased fetal movements are reported. Non-invasive prenatal tests (NIPT) made by choice excluded aneuploidies regarding chromosomes 21, 18, 13, 6, 9, X and Y and the more frequent microdeletion syndromes. He was born at 41 weeks by vaginal delivery. Birth parameters were: weight 3.940 kg (75th percentile), length 54 cm (90th percentile), head circumference 36 cm (75th percentile). Apgar scores were 9 and 10 at 1 and 5 minutes respectively. At birth he showed: right hand clenching, ulnar deviation of the hand, bilateral adducted thumb, overlapping fingers (F23 and F54 on right side; F23 and F43 on left side) and bilateral talipes calcaneovalgus. He also had microretrognathia. The first diagnostic hypothesis was of DA. Later, due to the finding of slight dilation of the aortic root on a required echocardiography, Loeys-Dietz syndrome was suspected. Head control was acquired at 3 months, walking with support at 14 months, walking alone at 15 months, babbling at 6 months and speech at 10 months. Bilateral clubfoot was treated according to Ponseti method, with serial manipulation and casting since 12 days, left Achilles tendon tenotomy at 3 months and again at 10 months, because of relapsing equinovarus. Then a foot abduction orthosis was introduced. Until 1 year and 4 months he was treated with a static splint at the right hand during the night and he is still using the foot abduction orthoses during the night. He was treated with physiotherapy at the hands and the feet. Echocardiography showed bicuspid aortic valve and mild-moderate dilatation of aortic root (1.9 cm at the level of the sinuses of Valsalva, Z score = +3.3 at 8 months; 24 mm at the level of the sinuses of Valsalva, Z-score = +3.7 at 1 year and 7 months. The following evaluations resulted normal: AOEs, fundus examination, ECG, cerebral ultrasound, abdominal ultrasound, X-ray of right hand. At the last clinical evaluation, at 1 year and 7 months, he showed: tall stature (length 90 cm, >97th percentile), weight 11.8 kg (50th percentile), macrocephaly (present also in the mother, head circumference 52 cm, >97th percentile, +4.3SD), frontal bossing, hypertelorism, blue sclerae, microretrognathia, bifid uvula and translucent skin especially on periorbital region and trunk. We also pointed out: bilateral ulnar deviation of the hand, single transverse palmar crease on the right hand, bilateral prominent calcaneus, left pes planus and overlapping toes (F21 and F23 on the left side; F12 and F32 on the right side).

<u>Genetic testing.</u> An NGS panel of 15 genes associated with aortopathies (*ACTA2, COL3A1, FBN1, FLNA, MAT2A, MFAP5, MYH11, MYLK, NOTCH1, PRKG1, SMAD3, TGFB2, TGFB3, TGFBR1, TGFBR2*) revealed the heterozygous frameshift variant c.1595dupA, p.(His532Glnfs*9) in the *TGFBR2* gene (NM_ 001024847.2). The same variant was present in the mother. This variant is unreported in scientific literature, disease databases and reference population databases [5,12,18-20]. The transcript is expected to escape nonsense-mediated RNA decay and generate a truncated protein, missing part of its kinase domain. Since this has already been established as a pathogenetic mechanism, the variant was classified as likely pathogenic [21,22]. An NGS panel of 11 genes associated with DA (*CHST14, ERBB3, FBN2, MYBPC1, MYH3, MYH8, PIEZO2, RAPSN, TNNI2, TNNT3, TPM2*), performed previously, revealed no alterations.

Family 7.

<u>Pt 12</u> is the first child of a non-consanguineous couple of Italian origins and the mother of 2 unaffected children. Her younger brother is healthy. She was evaluated for the first time at 3 years and 6 months. Pregnancy was characterized by miscarriage threats, oligohydramnios and decreased fetal

movement. Birth parameters were: weight 2.350 kg (<3rd percentile), length 49.5 cm (25th-50th percentile), head circumference 33 cm (10th-25th percentile). She showed jaundice, treated with phototherapy. At birth she showed in the upper extremity: internally rotated shoulders, axillary pterygium, extended elbows, flexion contracture of wrists, bilateral ulnar deviation of the hand, stiff fingers, adducted thumbs and partial cutaneous syndactyly of fingers. In the lower limbs she showed severe bilateral talipes equinovarus (more severe on the right side) and overlapping toes (F23). Reduction of limb muscular mass, absent flexion creases at the elbows, reduced flexion creases at the knees and joint stiffness were also noticed. Joint stiffness decreased over time with greater possibility of flexion movements. Clinical features were compatible with amyoplasia. Head control was acquired at 3 months, walking with support at 1 year, walking alone at 3 years, babbling at 5 months, speech at 1 year. She was treated with orthoses at the lower limbs and she underwent 2 surgical procedures at lower limbs (at 2 and 4 years respectively) and 3 surgical procedures at the hands (at 6, 7 and 8 years respectively). She uses orthopedic shoes until 17 years of age. Muscle biopsy showed fibroadipose replacement, mild changes in fiber cells and normal immunohistochemical analysis. At the last clinical evaluation, at 25 years during the first pregnancy, she showed: short stature (height 152.7 cm, <3rd percentile), weight 40 kg, head circumference 52.5 cm (3rd-10th percentile), short neck, down-sloping shoulders, flexion contracture of wrists and fingers, bilateral ulnar deviation of the hand, stiff fingers, adducted thumbs (> on left side) and overlapping toes (F23 and F43). She also showed reduction of limb muscular mass, scoliosis, axillary, antecubital and interdigital pterygia.

Genetic testing. Blood standard karyotype performed at birth was normal (46,XX).

Family 8.

Pt 13 is the second child of a non-consanguineous couple of Italian origins. An older brother is healthy. She was evaluated for the first time at 16 years and she is now 37 years old. Prenatal period was unremarkable. She was born at 39 weeks by caesarean section due to previous caesarean section. Birth parameters were: weight 3.100 kg (25th-50th percentile), length 48 cm (25th percentile), head circumference 34 cm (50th percentile). Apgar score was 1 at 1 minute. At birth, right clubfoot (pes valgus with metatarsus adductus) and enlarged female breast were noticed. At 2 days of life, she showed an episode of postprandial cyanosis. At 2 years her phenotype was similar to Williams syndrome, but FISH analysis for 7q11.23 region was normal. In childhood, mild ulnar deviation of right hand could be seen. At 16 years, camptodactyly was visible, with progressive worsening, together with clynodactyly of 3rd toe and overlapping toes. From her 26th year she showed signs of arthrosis, which has worsened since she turned 35. Trunk control was acquired at 9 months, walking with support at 15 months, walking alone at 20 months, babbling at 12 months, complete speech at 5 years. Due to speech delay she followed speech therapy. She was treated with serial cast at the right foot soon after birth. She was treated with physiotherapy and with corrective corset for scoliosis from 11 to 15 years. Due to worsening scoliosis she underwent 3 surgical procedures at 15, 26 and 34 years. She underwent 3 surgical procedures for the treatment of right lower limb: on her feet at 8 years for pes planus, on her toes at 19 years and on her ankle at 37 years. She is still using corrective corset, orthopedic shoes and orthotics. Echocardiography showed narrow right pulmonary artery. Abdominal ultrasound found gallbladder stones and calyceal

microlithiasis. X-ray of feet found fused and subluxate phalanges and from the age of 24 years elements of chondrolysis and reduction of calcium tone. Computational Tomography (CT) of feet showed pes planus and tarsal and metatarsal dysmorphisms. Chondropathy and arthrosis were also found in CT of the knees. X-rays of spine and CT of spine revealed progressive dorsolumbar scoliosis and arthrosis. MRI of ankles revealed cysts and diffuse degenerative phenomena. Lumbar dual-energy X-ray absorptiometry (DEXA) at 31 years was normal. Ophtalmological evaluation showed posterior embriotoxon, optic disc with blurred edges. The following evaluations showed normal results: cerebral CT and MRI. At the last clinical evaluation, at 35 years, she showed: short stature (height 146 cm, <3rd percentile), weight 52.5 kg (25th-50th percentile), head circumference 54 cm (25th-50th percentile, mean/+2SD), myopathic facies, sparse hair, bilateral epicanthus, bulbous nose, low hanging columella, microretrognathia, low-set ears, trismus, camptodactyly of fingers, cutaneous syndactyly between 2nd, 3th and 4th toes on the left side, mild cutaneous syndactyly between 2nd and 3th toes on the right side, clinodactyly of 2nd left toe, overlapping toes (F23 on the left side; F45 on the right side) and pes planus, scoliosis. On the hands she showed pterygia of digits.

Genetic testing. WES filtered for 28 genes associated with DA including *ECEL1* gene (*ACTA1, ADCY6, ALG3, BICD2, CHST14, CNTNAP1, DNM2, ECEL1, ERBB3, FBN2, FKBP10, GLE1, LGI4, MYBPC1, MYH3, MYH8, NALCN, PIEZO2, PIP5K1C, PLOD2, SYNE1, TNNI2, TNNT3, TOR1A, TPM2, VIPAS39, VPS33B, ZC4H2*) did not identify any pathogenic or likely pathogenic variant(s) in the tested genes. Blood standard karyotype, FISH for 7q11.23 region, FISH for subtelomeric regions, karyotype on fibroblasts, FISH of centromeric region of chromosome on fibroblasts (808 metaphases), performed previously,were normal. So, she does not have a molecular diagnosis.

References

1. PROVEAN. Available online: http://provean.jcvi.org/index.php (accessed on 16 November 2021).
2. SIFT. Available online: https://sift.bii.a-star.edu.sg/ (accessed on 16 November 2021).
3. Mutation Taster. Available online: http://www.mutationtaster.org/ (accessed on 16 November 2021).
4. PolyPhen-2. Available online: http://genetics.bwh.harvard.edu/pph2/ (accessed on 16 November 2021).
5. gnomAD v2.1.1.1. Available online: https://gnomad.broadinstitute.org/ (accessed on 16 November 2021).
6. Clarke, N.F.; Waddell, L.B.; Sie, L.T.; van Bon, B.W.; McLean, C.; Clark, D.; Kornberg, A.; Lammens, M; North, K.N. Mutations in TPM2 and congenital fibre type disproportion. *Neuromuscul Disord*. **2012**, *22(11)*, 955-8, doi: 10.1016/j.nmd.2012.06.002.
7. Marttila, M.; Lehtokari, V.L.; Marston, S.; Nyman, T.A.; Barnerias, C.; Beggs, A.H.; Bertini, E.; Ceyhan-Birsoy, O.; Cintas, P.; Gerard, M.; et al. Mutation update and genotype-phenotype correlations of novel and previously described mutations in TPM2 and TPM3 causing congenital myopathies. *Hum Mutat*. **2014**, *35(7)*, 779-90, doi: 10.1002/humu.22554.
8. Jin, J.Y.; Wu, P.F.; Fan, L.L.; Yu, F.; Li, J.J.; Fan, X.F.; Huang, H.; Zeng, L.; Tang, J.Y.; Xiang, R. A mutation of beta-tropomyosin gene in a Chinese family with distal arthrogryposis type I. *Int J Clin Exp Pathol*. **2017**, *10(11)*, 11137-11142.
9. Zhao, N.; Jiang, M.; Han, W.; Bian, C.; Li, X.; Huang, F.; Kong, Q.; Li, J. A novel mutation in TNNT3 associated with Sheldon-Hall syndrome in a Chinese family with vertical talus. *Eur J Med Genet*. **2011**, *54(3)*, 351-3, doi: 10.1016/j.ejmg.2011.03.002.
10. Beck, A.E.; McMillin, M.J.; Gildersleeve, H.I.; Kezele, P.R.; Shively, K.M.; Carey, J.C.; Regnier, M.; Bamshad, M.J. Spectrum of mutations that cause distal arthrogryposis types 1 and 2B. *Am J Med Genet A*. **2013**, *161A(3)*, 550-5, doi: 10.1002/ajmg.a.35809.
11. Shrimpton, A.E.; Hoo, J.J. A TNNI2 mutation in a family with distal arthrogryposis type 2B. *Eur J Med Genet*. **2006**, 49(2), 201-6, doi: 10.1016/j.ejmg.2005.06.003.
12. CLINVAR. Available online: https://www.ncbi.nlm.nih.gov/clinvar/ (accessed on 16 November 2021).
13. Shrimpton, A.E.; Hoo JJ. A TNNI2 mutation in a family with distal arthrogryposis type 2B. *Eur J Med Genet*. **2006**, *49(2)*, 201-6, doi: 10.1016/j.ejmg.2005.06.003.
14. Morgan, N.V.; Brueton, L.A.; Cox, P.; Greally, M.T.; Tolmie, J.; Pasha, S.; Aligianis, I.A.; van Bokhoven, H.; Marton, T.; Al-Gazali, L.; et al. Mutations in the embryonal subunit of the acetylcholine receptor (CHRNG)

cause lethal and Escobar variants of multiple pterygium syndrome. *Am J Hum Genet*. **2006**, *79(2)*, 390-5, doi: 10.1086/506256.

15. Vogt, J.; Morgan, N.V.; Rehal, P.; Faivre, L.; Brueton, L.A.; Becker, K.; Fryns, J.P.; Holder, S.; Islam, L.; Kivuva, E.; et al. L. CHRNG genotype-phenotype correlations in the multiple pterygium syndromes. J Med Genet. 2012, 49(1), 21-6, doi: 10.1136/jmedgenet-2011-100378.
16. Robinson, K.G.; Viereck, M.J.; Margiotta, M.V.; Gripp, K.W.; Abdul-Rahman, O.A.; Akins, R.E. Neuromotor synapses in Escobar syndrome. *Am J Med Genet A*. **2013**, *161A(12)*, 3042-8, doi: 10.1002/ajmg.a.36154.
17. Al Kaissi, A.; Ryabykh, S.; Ochirova, P.; Bouchoucha, S.; Kenis, V.; Shboul, M; Ganger, R.; Grill, F.; Kircher, S.G. Arthrogryposis is a descriptive term, not a specific disease entity: escobar syndrome is an Example. *Minerva Pediatr*. **2020**, doi: 10.23736/S0026-4946.20.05796-5. Epub ahead of print.
18. HGMD. Available online: http://www.hgmd.cf.ac.uk/ac/index.php (accessed on 16 November 2021).
19. LOVD. Available online: https://www.lovd.nl/ (accessed on 16 September 2021).
20. 1000 genomes. Available online: https://www.internationalgenome.org/ (accessed on 16 November 2021).
21. NMD Esc Predictor. Available online: https://nmdprediction.shinyapps.io (accessed on 16 November 2021).
22. Braverman, A.C.; Moon, M.R.; Geraghty, P.; Willing, M.; Bach, C.; Kouchoukos, N.T. Pregnancy after aortic root replacement in Loeys-Dietz syndrome: High risk of aortic dissection. *Am J Med Genet A*. **2016**, *170*(*8*), 2177-80, doi: 10.1002/ajmg.a.37694.
